# Nutrient Intake Evidence in the Pacific: A Scoping Review of Research Coverage, Challenges, and Opportunities

**DOI:** 10.1002/fsn3.71360

**Published:** 2025-12-28

**Authors:** Eliza Kitchener, Rachael Thurecht, Alannah Connors, Barnaby Dixson, Georgia Kafer

**Affiliations:** ^1^ School of Health University of the Sunshine Coast Sippy Downs Queensland Australia; ^2^ Australian Centre for Pacific Island Research University of the Sunshine Coast Sippy Downs Queensland Australia

**Keywords:** eating, micronutrients, nutrients, nutrition assessment, nutrition surveys, pacific islands, scoping review

## Abstract

Pacific Island Countries and Territories (PICTs) face a growing burden of malnutrition and diet‐related noncommunicable diseases. Yet, nutrient intake among Pacific populations remains unclear. This scoping review explores the available literature of macro‐ and micronutrient intakes among PICTs. Scholarly database searches and targeted web searches were conducted to capture sources reporting nutrient intake from 16 PICTs. Screening and data extraction were conducted by two independent reviewers, with results analyzed descriptively. Fifty‐one sources were included in the review. Papua New Guinea and Samoa were the most represented PICTs, while no relevant data from Nauru, Niue, and Tuvalu was identified. Dietary intake of infants and children was examined in 27% of sources, whereas women were the focus of investigation in only seven studies (13.7%). Common dietary assessment methods utilized were food frequency questionnaires, 24‐h recall surveys, and food records. Micronutrient intake was disproportionately reported in the literature compared with macronutrients (captured in 30% and 90% of sources, respectively). Our review highlights the scarcity of research on nutrient intake among PICTs, especially within nutritionally vulnerable groups—women and children. While nutrition research in the Pacific is increasing, alongside greater use of the Pacific Nutrient Database (PNDB) supporting analysis of region‐specific foods, the PNDB lacks nutrient composition data for key micronutrients (folate, iodine, and vitamin D), which were consequently the least‐reported nutrients in the literature. Expanding dietary intake research in the Pacific, particularly among under‐represented groups, women and children, is essential to identify nutritional gaps and inform evidence‐based nutrition policies and interventions.

## Graphical abstract

This scoping review mapped the available evidence on macro‐ and micronutrient intakes across 16 Pacific Island Countries and Territories (PICTs) in the Western Pacific (shown on the map). Of the 2021 sources captured and screened, 51 met inclusion criteria. Analysis revealed substantial knowledge gaps, including the absence of key nutrients from the regional nutrient database. Findings highlight both challenges and opportunities to expand nutrition research in the region.

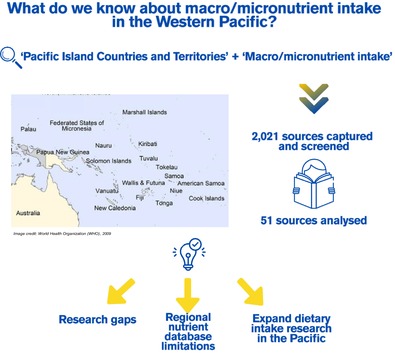

## 1 Introduction

Pacific Island Countries and Territories (PICTs) face a growing burden of malnutrition and dietary‐related noncommunicable disease (NCDs) (Blankenship et al. 2020; Sharp and Andrew 2021). Worsening malnutrition in the Western Pacific is linked to a transition in dietary behaviors observed across the region, shifting away from traditional diets, rich in fruits and vegetables, in favor of processed and packaged foods (Reeve et al. 2022; Sievert et al. 2019; Vogliano et al. 2020, 2021). This transition reflects broader changes to Western Pacific food systems, associated with globalization and shifting economic development in the region (Campbell 2015). Food systems are further impacted by climate change and extreme weather events (WHO Western Pacific Region 2015). Destruction of agricultural lands critical for subsistence farming and the loss of livelihoods in the aftermath of natural disasters poses a significant threat to accessing nutritious foods (Barnett 2020; Galanis et al. 1995; Scialabba 2009; Trudinger et al. 2023; WHO Western Pacific Region 2015). Pacific Island populations subsequently face a growing dependence on nutritionally low foods, exacerbating existing malnutrition and associated adverse health outcomes (FAO et al. 2024; Trudinger et al. 2023; WHO Western Pacific Region 2015).

The triple burden of malnutrition is well‐established among PICTs, with rising proportional mortality attributed to malnutrition over the last two decades (Peng et al. 2023). Nutrition‐associated NCDs, including cardiovascular disease and diabetes, are among the leading causes of death (Peng et al. 2023). Further, a recent WHO STEPS report identified that the prevalence of hypertension, hypercholesterolemia, and obesity has significantly increased in the preceding decade among most PICTs nations surveyed (Reeve et al. 2022). In several Pacific Islands, women face a greater burden of overweight and obesity than men (Reeve et al. 2022; Vanuatu Ministry of Health 2022). Improvements in rates of anemia among women in the Pacific have also stagnated since 2000, contributing to greater maternal and perinatal mortality risk (FAO 2024). Childhood stunting associated with malnutrition is also a significant health concern among PICTs, with prevalence rates among under 5‐year‐olds greater than those of the East Asia and Pacific region (18% compared with 11%, respectively) (UNICEF 2017). There have been calls for further nutrition research in PICTs as little is known about their nutrient consumption (Blankenship et al. 2020; FAO 2024; Reeve et al. 2022), despite the growing burden of NCDs and nutritional deficits in the Western Pacific.

This scoping review aimed to identify the available literature pertaining to quantitative macronutrient and micronutrient intakes among PICT populations. Our focus on nutrient intake of Pacific Islander peoples was identified as a priority to ascertain the availability of dietary data that can inform targeted health initiatives, policy development and future dietary assessments conducted among Pacific Island populations. Understanding trends pertaining to quantitative measurements of nutrient intake is important as this knowledge can provide detailed insight into nutritional adequacy against dietary requirements (Bailey 2021). In this study we review current knowledge and map the common methods of estimating nutrient intake data used in the Western Pacific. These findings will guide future nutritional research in PICTs to address the burden of malnutrition and associated poor health outcomes experienced among Pacific Island peoples.

### 1.1 Key Messages

- PICTs are facing increased malnutrition and dietary‐related NCDs associated with a dietary transition away from traditional diets to greater consumption of processed foods.
- Despite the growing burden of NCDs and nutritional deficits in the Pacific, little is known about the maternal and child triple burden of malnutrition and the nutrient intake of the broader population.
- This scoping review mapped existing literature on macro‐ and micronutrient dietary intake among PICT populations to inform health initiatives, policy development, and future research.

## 2 Materials and Methods

The broad approach of a scoping review is beneficial to indicate the volume and quality of available literature within a research field (Munn et al. 2018). This approach is critical for PICT regions where a significant proportion of available data is generated by global and local humanitarian or government bodies rather than through peer‐reviewed research. For this review, the framework developed by Arksey and O'Malley (2005) was followed by (1) identifying research question(s), (2) identifying relevant studies, (3) selecting relevant studies, (4) charting the data, and finally, (5) collating, summarizing, and analyzing findings (Arksey and O'Malley 2005). Collation and summary of findings is presented in narrative form in accordance with the guidelines prescribed by the Preferred Reporting Items for Systematic Reviews and Meta‐Analyses extension for Scoping Reviews (PRISMA‐ScR) (Tricco et al. 2018). The full protocol is available on Open Science Framework (https://doi.org/10.17605/OSF.IO/6EGTM).

### 2.1 Inclusion and Exclusion Criteria

This scoping review was guided by two key themes, (1) dietary intake of macro‐ and micronutrients, and (2) PICTs. The focus of the review was to explore dietary intake, rather than nutritional status, as the latter can be influenced by a variety of metabolic factors (Picó et al. 2019), thus, sources which included biochemical analyses were only eligible for inclusion if macro or micronutrient dietary intake was also reported. Literature was eligible for inclusion in the study if quantified macro and/or micronutrient intake was reported. Sources were excluded if only whole foods or food groups were reported (i.e., macro/micronutrient intake not reported). The population of interest included all people residing in PICTs. The 16 PICTs referred to in this study are mostly comprised of Pacific Island Small Developing States (PSIDS), as determined by the UN Department of Economic and Social Affairs (Otto 2014). American Samoa and Tokelau, although not recognized as PSIDS, were also included in this review as they share geographic, cultural, and socioeconomic similarities with neighboring Pacific Islands. The PICTs included in this review were American Samoa, the Cook Islands, the Federated States of Micronesia (FSM), Fiji, Kiribati, the Republic of Marshall Islands (RMI), Nauru, Niue, Palau, Papua New Guinea (PNG), Samoa, the Solomon Islands, Tokelau, Tonga, Tuvalu, and Vanuatu. Culture, socioeconomic status, and environmental factors heavily influence dietary behaviors (Enriquez and Archila‐Godinez 2022). Given this, data pertaining to any peoples residing in any PICTs was considered relevant regardless of cultural background and ethnicity. However, it is important to note that studies focused on Pacific Island peoples not residing within a PICT region of interest were excluded. A broad range of source types were considered for review, including quantitative and mixed‐methods study designs, systematic reviews, gray literature, books, theses, and conference papers. Qualitative studies were excluded, as were papers with no full text or methodology available, papers not published in English, reference works and blogs.

### 2.2 Search Strategy

The search strategy was designed to capture all available published literature reporting macronutrient and micronutrient intakes in PICTs. Several databases were searched including PubMed, Scopus, CINHL, ProQuest, Web of Science, and Google Scholar. No date range was set for literature searches. A targeted gray literature search was conducted, guided by The Pacific Community Statistics for Development Division (SPC SDD) Census and Survey Calendar (SPC Statistics for Development Division 2024), which lists census and survey reports completed in PICTs between 1990 to present. Initial database and gray literature searches were completed in August 2023. The literature search process was repeated on the 23rd of August 2024 to capture additional literature published over the preceding 12‐month period.

### 2.3 Screening and Data Analysis

All literature retrieved from the database and targeted searches were collated and uploaded to Covidence (covidence.org) for screening and eligibility assessment by two independent reviewers. Literature was initially screened by title and abstract to determine relevancy. Full‐text articles were then reviewed to assess eligibility for inclusion in the review. Data were extracted from the final pool of included literature using Covidence. Conflicts that arose between the two reviewers were discussed and, where necessary, resolved through consultation with a third researcher. Following extraction, data were exported to Microsoft Excel for collation and descriptive analysis.

## 3 Results

**FIGURE 1:**
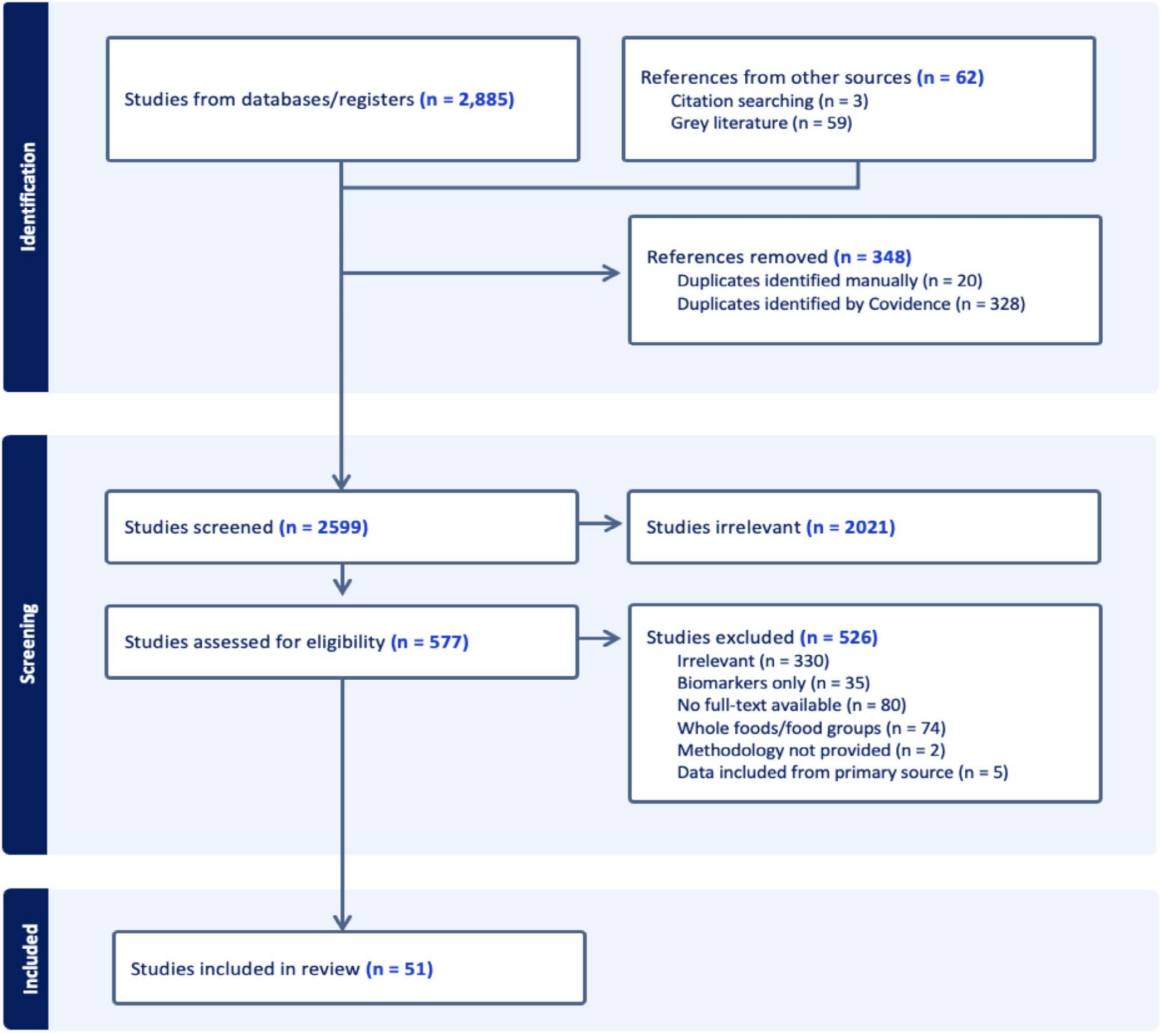
PRISMA flow chart demonstrates the process of source identification, screening, and exclusion following PRISMA guidelines.

The literature search yielded 2961 sources. After removal of duplicates (*n* = 351) and irrelevant articles at initial screening (*n* = 2021) a total of 577 sources were reviewed for eligibility. Full texts were then evaluated for eligibility and excluded if considered irrelevant to nominated key concepts, if no full text was available, or if the data was already extracted from the primary (Figure 1). A total of 51 sources were included in our final review (Table 1).

**TABLE 1:** Literature summary table: Description of sources included in the review: Country where the research took place, source type, characteristics of the study cohort, research aims, method of dietary assessment used, and the macro‐ and micronutrients studied.

Source	Location	Source type	Cohort characteristics	Aims	Dietary collection method	Nutrients studied
Some Implications of the Diet of Children in American‐Samoa (1994); Binden, J. R.	American Samoa	Peer‐reviewed journal article	American Samoans (*n* = 62) Age: Children (5–7 years) Sex: Male (*n* = 31) and female (*n* = 31)	To describe and interpret the nutrient intake.	24 h recall	Fats, protein, calcium, iron, phosphorous, niacin, riboflavin, thiamine, vitamin A, vitamin C
Vitamin A intake and factors influencing it among children and caretakers in Kosrae, Micronesia (2005); Englberger et al.	Federated States of Micronesia (FSM); Kosrae	Peer‐reviewed journal article	Micronesians FFQ = Children (*n* = 267); Caretakers (*n* = 132) 24 h recall = Children (*n* = 65); Caretakers (*n* = 65) Age: Children (2.5–6.5 years); Adults (17–74 years) Sex: Children (172 M/160F); Adults (1 M/196 F)	(1) To develop an appropriate tool for measuring and monitoring dietary VA among preschool children and their caretakers; (2) assess and describe dietary VA intake; and (3) investigate relationships between dietary intake and factors influencing it.	24 h recall (x3 non‐consecutive days); 7‐day FFQ	Fats, protein, vitamin A
Evaluation of a traditional food for health intervention in Pohnpei, Federated States of Micronesia (2010); Kaufer et al.	Pohnpei (FSM)	Peer‐reviewed journal article	Micronesians (24 h recall *n* = 26; FFQ *n* = 40) Age: Adults^a^ Sex: Female	Assess changes in diet and health that may have been impacted by a two‐year intervention in one Pohnpeian community.	24 h recall (x2 non‐consecutive days); 7‐day FFQ At baseline 2005 & follow‐up 2007	Carbohydrates, fats, protein, calcium, iron, vitamin A, vitamin C
Fiji National Nutrition Survey: Main report (2007); Schultz et al.	Fiji	Gray literature	Fijians (FFQ *n* = 1693); (24 h recall *n* = 2272) Age: Infants (< 2 years), adolescents—adults (≥ 15 years) Sex: Male and female^a^ Subset of population: WRA (15–44 years) not breastfeeding or lactating^a^	Collect information on national nutritional health based on food and nutrient intakes.	24 h recall; FFQ	Carbohydrates, fats, protein, calcium, iron, potassium, zinc, thiamine, vitamin A, vitamin C
Correspondence between human diet, body composition and stable isotopic composition of hair and breath in Fijian villagers (2009); Hedges et al.	Fiji	Peer‐reviewed journal article	Fijians (*n* = 20) Age: Adults (19–65 years) Sex: Female Subset of population: WRA not breastfeeding or lactating (*n* = 20)	To describe the relationship between diet, and hair and breath isotopic composition.	Food record (14‐day)	Carbohydrates, fats, protein
Food offerings on board and dietary intake of European and Kiribati seafarers—cross‐sectional data from the seafarer nutrition study (2018); Zyriax et al.	Kiribati	Peer‐eviewed journal article	I‐Kiribati (*n* = 48); Europeans (*n* = 33) Age: Adults (20–64 years) Sex: Male	To analyze food quantity and quality on merchant vessels in relation to current recommendations, to compare individual nutrition intake, food preferences and satisfaction, to identify unhealthy dietary habits.	24 h recall	Carbohydrates, fats, fiber, protein, iodine, sodium, folate,^b^ folic acid, riboflavin, thiamine, vitamin D, vitamin E
Quantitative estimates of dietary intake in households of South Tarawa, Kiribati (2019); Eme et al.	Kiribati	Peer‐reviewed journal article	I‐Kiribati (*n* > 161) Recall: *n* = 161 (43 M/118F) Food record: *n* = 29^a^ (sub‐sample reporting on behalf of 8 households) Age: Children—adults (4–75 years) Sex: Male and female^a^	To quantitatively assess dietary patterns, food intake, and dietary diversity of adult householders in South Tarawa, Kiribati.	24 h recall; Food record (3‐day, weighed)	Protein, calcium, iron, magnesium, potassium, sodium, zinc, riboflavin, thiamine, vitamin A, vitamin C
Are households in Kiribati nutrition secure? A case study of South Tarawa and Butaritari (2020); Eme et al.	Kiribati	Peer‐reviewed journal article	I‐Kiribati (*n* = 541) Recall: *n* = 468 Food record: *n* = 73 Age: Infants—adults (1–65 years) Sex: Male (*n* = 187) and female (*n* = 354)	To measure the nutrient intake, food variety and diet diversity of adult householders in South Tarawa and Buta Ritari, Kiribati.	24 h recall; food record (3‐days, researcher direct observation, weighed)	Carbohydrates, fats, protein, calcium, iron, magnesium, potassium, sodium, zinc, niacin, riboflavin, thiamine, vitamin A, vitamin C
Modeling thiamine fortification: a case study from Kuria atoll, Republic of Kiribati (2021); Green et al.	Kiribati	Peer‐reviewed journal article	I‐Kiribati (*n* = 155) Age: Adults (20–48 years) Sex: Male (*n* = 90), female (*n* = 102) Subset of population: Pregnant women (*n* = 17), lactating women (*n* = 45), WRA (*n* = 42)	To design a food fortification strategy to improve thiamine intakes in Kuria.	24 h recall (x2 consecutive days)	Thiamine
Food consumption in Kiribati: Based on analysis of the 2019/20 Household Income and Expenditure Survey (2021); Troubat and Sharp	Kiribati	Gray literature	I‐Kiribati (*n* = 2182 households) Age: Not reported. Sex: Male and female^a^	To provide indicators on food security and food consumption in Kiribati, to inform the development of policies aiming to improve the food security status of I‐Kiribati.	7‐day diet history (HIES data)	Carbohydrates, fats, fiber, protein, calcium, iron, riboflavin, thiamine, vitamin A, vitamin B12, vitamin C
Nutrition Study in Micronesia (1954); Murai, M.	Republic of Marshall Islands (RMI)	PhD dissertation	Micronesians (*n* = 614) Age: Infants—adults (< 1–70 years) Sex: Male (*n* = 290), female (*n* = 324) Subset of population: Pregnant (*n* = 4), lactating (*n* = 38)	To study dietary habits and nutritional status of inhabitants, and the nutrient composition of basic plant and animal foods, to develop effective methods of gathering nutrition information necessary for promoting educational and developmental programs.	Food record (3–7 days, participant‐recorded and researcher direct observation, weighed)	Fats, protein, calcium, iron, niacin, phosphorous, riboflavin, thiamine, thiamine, vitamin A, vitamin C
Dietary intake and nutritional status in Marshallese children (2001); Gammino, V.	RMI	PhD dissertation	Marshallese (*n* = 150) Age: Children (< 6 years) Sex: Male (*n* = 79), female (*n* = 71)	Describe the nutritional status of infants and young children and examine the relationships between these outcomes and the environmental and social factors commonly associated with poor nutritional status, particularly variation in degree of urbanization and modernization.	24 h recall (x3 non‐consecutive days)	Carbohydrates, fats, protein, iron
Poverty, food consumption, labour and Household Income and Expenditure in the Marshall Islands; a compendium of analysis of the 2019/20 Household Income and Expenditure Survey (2022b); The Pacific Community (SPC)	RMI	Gray literature	Marshallese (*n* = 870 households) Age: Not reported Sex: Male and female^a^	To better understand the socioeconomic situation of all Marshall Islanders.	Diet history (7‐day) (HIES data)	Carbohydrates, fats, protein, calcium, iron Riboflavin, thiamine, vitamin A, vitamin B12, vitamin C
Health Effects of Modernization in Palau (1973); Labarthe et al.	Palau	Peer‐reviewed journal article	Palauan (*n* = 261) Age: Adults (20–60+ years) Sex: Male (*n* = 109), female (*n* = 152)	To describe and analyze sociocultural indices of modernization and several measures of health status among three groups of adult Palauans.	24 h recall	Carbohydrates, fats, protein
Dietary and Disease Patterns among Micronesians (1970); Hankin et al.	Palau	Peer‐reviewed journal article	Palauan (*n* = 201), Guamians (*n* = 201), Rota peoples (*n* = 201), Californians (*n* = 201) Age: Adults (0–60+ years) Sex: Male (*n* = 165), female (*n* = 214)	To evaluate the effects of varying intensity of Western culture on the health of Pacific Islanders, through comparing dietary surveys, nutrient intakes and disease patterns of the four groups.	24 h recall	Carbohydrates, fats, protein
Nutritional and anthropometric assessment of a sample of pregnant women and young children in Palau (2000); Pobocik et al.	Palau	Peer‐reviewed journal article	Palauan (*n* = 57); (pregnant women (*n* = 26), children (*n* = 31)) Age: Children (1–6 years); Adults (19–29 years) Sex: Male (*n* = 17), female (*n* = 40) Subset of population: pregnant women (*n* = 26)	To generate baseline data and describe the nutritional and anthropometric profiles of a purposively selected group of pregnant women and children.	24 h recall	Carbohydrates, fats, protein, calcium, folate, iron, magnesium, potassium, sodium, zinc, thiamine, vitamin A, vitamin B6, vitamin B12, vitamin C, vitamin E
Urbanization, diet, and potential health effects in Palau (1972); Hankin and Dickinson	Palau	Peer‐reviewed journal article	Palauan (*n* = 261) Age: Adults (20–60 + years) Sex: Male (*n* = 109), female (*n* = 152)	To describe the dietary patterns of the three areas and propose hypotheses for testing potential health effects.	24 h recall	Carbohydrates, cholesterol, fats, protein, calcium, iron, niacin, riboflavin, thiamine, vitamin A, vitamin C
Energy and nutrient intake and energy expenditure of 204 New Guinean adults (1974); Norgan et al.	Papua New Guinea (PNG)	Peer‐reviewed journal article	Papua New Guineans (*n* = 204) Age: Adults (18–47 years) Sex: Male (*n* = 94), Female (*n* = 110) Subset of population: Pregnant women (*n* = 16), lactating women (*n* = 52)	To describe the nutrient and energy intake of adults living in a coastal village (Kaul) and in a highland village (Lufa).	Food record (5–7 days, researcher direct observation, weighed)	Carbohydrates, fats, protein
Food intake, its relationship to body weight and age, and its apparent nutritional adequacy in New Guinean children (1975); Ferro‐Luzzi et al.	PNG	Peer‐reviewed journal article	Papua New Guineans (*n* = 482) Age: Infants – adolescents (1–18 years) Sex: Male (*n* = 231), female (*n* = 251)	Study the dietary intakes of children in Papua New Guinea living in two contrasting environments near the coast (Kaul) and in a highland region (Lufa).	Food record (researcher direct observation, 5–7 consecutive days, weighed)	Carbohydrates, fats, protein
Nutritional Status of Papua New Guinea Highlanders (1981); Okuda et al.	PNG	Peer‐reviewed journal article	Papua New Guineans (*n* = 18) Age: Adults (20–40 years) Sex: Male	To determine how Papua New Guinean peoples are nutritionally adapted to a low protein diet.	24 h recall (2–3 consecutive days)	Carbohydrates, fats, fiber, protein, ash, calcium, iron, phosphorous, sodium, Niacin, riboflavin, thiamine, vitamin A, vitamin C
The Seasonal Factors Influencing Child Malnutrition on the Nembi Plateau, Papua New Guinea (1986); Crittenden and Baines	PNG	Peer‐reviewed journal article	Papua New Guineans (*n* = 43) Age: Infants – children (< 5 mo–5 years) Sex: Male and female^a^	To monitor the nutritional status of a sample of children from one clan on the Nembi Plateau.	24 h recall (x3 per month, over 12 months, food checklist to prompt memory)	Carbohydrates, protein, fats
An Ecological Analysis of Child Malnutrition in an Abelam Community, Papua New Guinea (1987); Tyson, D.	PNG	PhD dissertation	Papua New Guineans (*n* = 40) Age: Children (< 5 years) Sex: Male (*n* = 19), female (21)	To provide understanding of the ultimate causes of child malnutrition, in what appear to be conditions of relative food abundance.	24 h recall (x8 repeated, 6‐weekly)	Protein
Growth in children from the Wosera subdistrict, Papua New Guinea, in relation to energy and protein intakes and zinc status (1991); Gibson et al.	PNG	Peer‐reviewed journal article	Papua New Guineans (*n* = 67) Age: Infants–children (6 mo–10 years) Sex: Male (*n* = 32), female (*n* = 35)	Examine the growth children living in the Wosera Subdistrict of PNG, in relation to their energy and protein intakes, and zinc status.	24 h recall (x2 repeated 1 week apart)	Carbohydrates, fiber, protein, fats, calcium, iron, zinc, phytate
Dietary and Nutrient Intakes of 25 Ningerum (New Guinea) Adult Males at Two Times of the Year (1992); Ulijaszek, S. J.	PNG	Peer‐reviewed journal article	Papua New Guineans (*n* = 25) Age: Adults (18–40 years) Sex: Male	To examine the assumption, that dietary intakes are sufficiently homogeneous for dietary studies of short duration to be adequate for accurate assessment of group nutrient intake among Papua New Guinean adults.	Food record (5‐days, researcher direct observation, weighed)	Carbohydrates, fats, protein, calcium, iron, niacin, riboflavin, thiamine, vitamin A, vitamin C
Diet in an urban Papua New Guinea population with high levels of cardiovascular risk factors (1996b); Hodge et al.	PNG	Peer‐reviewed journal article	Papua New Guineans (*n* = 285, completed both 24‐h recall and FFQ) Age: Adults (≥ 25 years) Sex: Male (*n* = 148), female (*n* = 137)	To report an example of the diet in an urban group of adults with high levels of cardiovascular risk factors.	24 h recall; FFQ	Carbohydrates, fats, fiber, protein
A Case–Control Study of Diet in Newly Diagnosed NIDDM in the Wanigela People of Papua New Guinea (1996a); Hodge et al.	PNG	Peer‐reviewed journal article	Papua New Guineans (*n* = 285, completed both 24‐h recall and FFQ) Age: Adults (≥ 25 years) Sex: Male (*n* = 148), female (*n* = 137)	Case–control study to investigate the association between diet and newly diagnosed type II diabetes in the Wanigela people of Papua New Guinea.	24 h recall; FFQ	Carbohydrates, fats, fiber, protein
Diet among the Huli Papua New Guinea Highlands when they were influenced by the extended rainy period (1999); Umezaki et al.	PNG	Peer‐reviewed journal article	Papua New Guineans (*n* = 21 households) Age: Adults^a^ Sex: Male and female^a^	To study the impact of extended rainy periods on nutritional intake, comparing 2 communities in the Huli region.	Food record (7‐days, researcher direct observation, weighed)	Fats, protein
Cross‐sectional dietary deficiencies among a prison population in Papua New Guinea (2013); Gould et al.	PNG	Peer‐reviewed journal article	Papua New Guineans (*n* = 161) Recall: *n* = 161 (148 prisoners/9 guards) FFQ: *n* = 9 (guards only) Age: Adults (≥ 23 years) Sex: Male	Investigate the dietary adequacy of prisoners of Beon Prison, PNG, in response to a report of possible nutritional deficiency.	24 h recall (weighed); FFQ	Carbohydrates, fats, protein Calcium, iron, magnesium, potassium, sodium, zinc, folate, riboflavin, thiamine, vitamin A, vitamin B6, vitamin B12, vitamin C, vitamin E
Stagnant Stunting Rate despite Rapid Economic Growth‐An Analysis of Cross Sectional Survey Data of Undernutrition among Children under Five in Papua New Guinea (2016); Hou, X.	PNG	Peer‐reviewed journal article	Papua New Guineans (*n* = 3057) Age: Children (< 5 years) Sex: Male and female^a^	To investigate undernutrition and factors which are significantly associated with undernutrition in PNG to guide nutrition‐related policies.	Food record (14‐day); Household stock data (day 1, day 14) (HIES data)	Protein
Iodine status of children and knowledge, attitude, practice of iodized salt use in a remote community in Kerema district, Gulf province, Papua New Guinea (2018); Goris et al.	PNG	Peer‐reviewed journal article	Papua New Guineans (*n* = 289) Age: Children (6–12 years) Sex: Male (*n* = 175), female (*n* = 114)	Assess the iodine status of children, and knowledge, attitudes and practice relating to use of iodised salt in a remote community in Kotidanga area, Gulf province, Papua New Guinea.	Discretionary intake of salt	Iodine
Iodine status of non‐pregnant women and availability of food vehicles for fortification with iodine in a remote community in Gulf province, Papua New Guinea (2019); Goris et al.	PNG	Peer‐reviewed journal article	Papua New Guineans (*n* = 284) Age: WRA (15–45 years) Sex: Female Subset of population: Non‐pregnant and non‐lactating women only (*n* = 284).	To re‐assess the iodine status of non‐pregnant women of reproductive age, the availability of commercial salt and the extent to which it was iodised, and the availability of other industrially processed foods that might be fortified with iodine.	Discretionary intake of salt	Iodine
Dietary intake changes associated with post‐cyclone food aid in Western Samoa (1995); Galanis et al.	Samoa	Peer‐reviewed journal article	Samoans (*n* = 147) Age: Adults (28–50 years) Sex: Male (*n* = 72), female (*n* = 75)	To examine dietary intake responses to a food aid program in Western Samoa and provide the first empirical suggestion that food aid programs may have a lasting effect on the food preferences of recipients.	FFQ (repeated pre‐ and post‐cyclone)	Carbohydrates, fats
Child, maternal and household‐level correlates of nutritional status: a cross‐sectional study among young Samoan children (2017); Choy et al.	Samoa	Peer‐reviewed journal article	Samoans (*n* = 305) Age: Children (2–4.99 years) Sex: Male (*n* = 157), female (*n* = 148)	Document the prevalence, coexistence and correlates of stunting, overweight/obesity and anemia in Samoan children.	FFQ	Carbohydrates, fats, fiber, protein, calcium, iron, sodium, potassium, niacin, vitamin A, vitamin C, vitamin E
Nutrient intake among Samoan children aged 2–4 years in 2015 (2018); Choy et al.	Samoa	Peer‐reviewed journal article	Samoans (*n* = 305) Age: Children (2–4.99 years) Sex: Male and female^a^	To examine the adequacy of macro‐ and micronutrient intake among Samoan children.	FFQ	Carbohydrates, fats, protein, calcium, iron, sodium, potassium, vitamin A, vitamin C, vitamin E
Developing a context‐specific nutrient profiling system for food policy in Samoa (2019); Reeve et al.	Samoa	Peer‐reviewed journal article	Samoans^a^ Age: Not reported Sex: Male and female^a^	To develop a transparent system for defining ‘less healthy’ foods to underpin effective policy to reduce noncommunicable diseases in Samoa, replacing a fatty‐meat ban lifted for accession to the WTO.	14‐day record household food expenditure/subsistence (HIES data)	Fats, protein, iron, sodium, vitamin A
Effect of maternal nutrient intake during 31–37 weeks gestation on offspring body composition in Samoa (2020); Arslanian et al.	Samoa	Peer‐reviewed journal article	Samoans (*n* = 107) Age: Adults (≥ 18 years) Sex: Female Subset of population: Pregnant women (*n* = 107)	To examine dietary macro and micronutrient intake of Samoan women during the third trimester of pregnancy and associations with infant body composition.	FFQ	Carbohydrates, fats, fiber, protein, calcium, iron, sodium, potassium, vitamin A, vitamin C, vitamin E
Sex differences in the associations of physical activity and macronutrient intake with child body composition: A cross‐sectional study of 3‐to 7‐year‐olds in Samoa (2020); Thompson et al.	Samoa	Peer‐reviewed journal article	Samons (*n* = 83) Age: Children (3–7 years) Sex: Male (*n* = 40), female (*n* = 43)	To describe body composition among Samoan children and determine sex‐specific associations among dietary intake, physical activity, and body composition.	FFQ	Carbohydrates, fats, protein
Food security and food consumption in Samoa based on the analysis of the 2018 Household Income and Expenditure Survey (2020); Troubat et al.	Samoa	Gray literature	Samoans (*n* = 2812 households) Age: Not reported. Sex: Male and female^a^	To identify the incidence of hardship and poverty, and to update the consumer price index, among other indicators, derived from analysis of the food data and FIES data collected in the 2018 HIES.	Food record (14‐day); household stock data (HIES data)	Carbohydrates, fats, protein, calcium, iron, riboflavin, thiamine, vitamin A, vitamin B12, vitamin C
Is overweight or obesity associated with anemia in children? Follow‐up of Samoans in the Ola Tuputupua'e “Growing Up” study (2022); Choy et al.	Samoa	Peer‐reviewed journal article	Samoans (*n* = 197) Age: Children (2–4 years) Sex: Male (*n* = 98), female (*n* = 99)	Examine whether overweight/obesity at 2–4 is associated with anemia among 3.8–6‐year‐old Samoan children.	FFQ	Calcium, iron, vitamin C
Potential Dietary Contributions from Rice and Wheat Flour Fortification in the Solomon Islands: Results From the 2012–2013 Household Income and Expenditure Survey (2019); Imhoff‐Kunsch et al.	Solomon Islands	Peer‐reviewed journal article	Solomon Islanders (*n* = 3122 households) Age: Not reported. Sex: Male and female^a^	To determine whether fortified rice (proposed) and fortified wheat flour potentially benefit women of reproductive age.	14‐day food expenditure record (HIES data)	iron, zinc, folic Acid, thiamine
Food consumption in Solomon Islands: Based on the analysis of the 2012/13 Household Income and Expenditure survey (2021); Troubat et al.	Solomon Islands	Gray literature	Solomon Islanders (*n* = 4364 households) Age: Not reported. Sex: Male and female^a^	To provide indicators on food security and food consumption in the Solomon Islands, to inform the development of policies aiming to improve the food security status of the population.	14‐day food record (HIES data)	Carbohydrates, fats, protein, calcium, iron, riboflavin, thiamine, vitamin A, vitamin B12, vitamin C
Dietary agrobiodiversity for improved nutrition and health outcomes within a transitioning indigenous Solomon Island food system (2021); Vogliano et al.	Solomon Islands	Peer‐reviewed journal article	Solomon Islanders (*n* = 30) Age: Adolescents – adults (15–49 years) Sex: Female (*n* = 30) Subset of population: Non‐pregnant or lactating WRA (*n* = 30)	To evaluate the contribution of agrobiodiversity from the local food system to diet quality.	24 h recall (x2 non‐consecutive days)	Carbohydrates, fats, fiber, protein, calcium, iron, magnesium, phosphorous, sodium, potassium, niacin, riboflavin, thiamine, vitamin A, vitamin C, zinc
Assessing Diet Quality of Indigenous Food Systems in Three Geographically Distinct Solomon Islands Sites (Melanesia, Pacific Islands) (2020); Vogliano et al.	Solomon Islands	Peer‐reviewed journal article	Solomon Islanders (*n* = 94) Age: Adolescents – adults (15–49 years) Sex: Female (*n* = 94) Subset of population: Non‐pregnant or lactating WRA (*n* = 94)	To assess nutrition transitions and diet quality by comparing three geographically unique rural and urban indigenous Solomon Islands populations.	24 h recall (x2 non‐consecutive days)	Carbohydrates, fats, fiber, protein, calcium, iron, zinc, magnesium, sodium, potassium, riboflavin, thiamine, vitamin A, vitamin C
The Tokelau Island Migrant Study: Serum Lipid Concentrations in Two Environments (1981); Stanhope et al.	Tokelau	Peer‐reviewed journal article	Tokelauan (*n* = 18 Fakaofo households; Taupo sample size not reported) Age: Not reported. Sex: Male and female^a^	To describe the distribution of serum lipids in Tokelauans aged 15 year and over, residing in Tokelau in 1976 or in New Zealand 1975–77.	24 h recall; Food record (researcher direct observation, 7‐day, weighed)	Carbohydrates, fats, protein
Energy and Nutrient Intake of Tongan Adults Estimated by 24‐h Recall: The Importance of Local Food Items (2011); Konishi et al.	Tonga	Peer‐reviewed journal article	Tongans (*n* = 34) Age: Adults (40–59 years) Sex: Male (*n* = 15), female (*n* = 19)	To estimate Tongans' energy and nutrient intakes and food sources.	24 h recall (x2 7‐consecutive days)	Carbohydrates, fats, protein, calcium, iron, riboflavin, thiamine, vitamin A, vitamin C
Vanuatu Dietary Study 1985 Summary Report (1993); Badcock et al.	Vanuatu	Gray literature	Ni‐Vanuatu (*n* = 431, completed both 24‐h recall and FFQ) Age: Adults^a^ Sex: Male (*n* = 267), female (*n* = 74)	To collect information on the dietary patterns of ni‐Vanuatu peoples, to correlate with the prevalence of noncommunicable disease and associated risk factors.	24 h recall; FFQ	Carbohydrates, fats, protein, calcium, iron, niacin, riboflavin, thiamine, vitamin A, vitamin C
Identifying the household factors, and food items, most important to nutrition in Vanuatu (2015); Martyn et al.	Vanuatu	Gray literature	Ni‐Vanuatu (*n* = 3975 households) Age: Infants‐adults (0–65+ years) Sex: Male and female^a^	To identify households most at risk of poor nutrition outcomes in Vanuatu, using microdata from the HIES (2010).	14‐day food expenditure record (HIES data)	Fats, protein, iron, sodium, vitamin A
Dietary intake of modernizing Samoans: Implications for risk of cardiovascular disease (1999); Galanis et al.	American Samoa; Samoa	Peer‐reviewed journal article	American Samoans (*n* = 455), Samoans (*n* = 491) Age: Adults (25–55 years) Sex: Male (*n* = 435), female (*n* = 511)	To describe the dietary intake, with emphasis on nutrients conventionally related to risk factors for cardiovascular disease.	24 h recall	Carbohydrates, fats, protein, calcium, potassium, sodium
Dietary Patterns Are Associated with Metabolic Syndrome in Adult Samoans (2009); Di Bello et al.	American Samoa; Samoa	Peer‐reviewed journal article	American Samoans (*n* = 723); Samoans (*n* = 785) Age: Adults (≥ 18 years) Sex: Male (*n* = 672), female (*n* = 836)	To describe dietary patterns to identify neo‐traditional and modern eating patterns and to relate these patterns to the presence of metabolic syndrome.	7‐day FFQ	Fats
Cholesterol, coconuts, and diet on Polynesian atolls: a natural experiment: the Pukapuka and Tokelau Island studies (1981); Prior et al.	Cook Islands; Tokelau	Peer‐reviewed journal article	Cook Islander (*n* = 165), Tokelauan (*n* = 77) Age: Adults (25–54 years) Sex: Male (*n* = 113), female (*n* = 129)	To investigate the relative effects of saturated fat and dietary cholesterol in determining serum cholesterol levels.	24 h recall (7 consecutive days)	Carbohydrates, fats, protein
Food Group, Macronutrient Intake, and Metabolic Status in the US‐Affiliated Pacific's Children's Healthy Living (CHL) Program (2022); Novotny et al.	American Samoa; Palau; RMI; FSM	Peer‐reviewed journal article	Marshall Islander (*n* = 191), American Samoan (*n* = 588), Palauan (*n* = 166), Micronesian (*n* = 625), Alaskan (*n* = 308), Guamanian (*n* = 666), Hawaiian (*n* = 430) Age: Children (2–8 years) Sex: Male (*n* = 1795), female (*n* = 1725)	To describe food group and macronutrient intakes of NHOPI children in the US‐Affiliated Pacific Island region, overall and by jurisdiction, income level, and metabolic status.	Food record (2‐days, measured)	Carbohydrates, fats, protein

Abbreviations: FFQ, food frequency questionnaire; FSM, Federated States of Micronesia; HIES, household income and expenditure survey; PNG, Papua New Guinea; SPC, The Pacific Community; WRA, women of reproductive age.

^a^ Specific detail not reported.

^b^ NB: folic acid/folate used interchangeably in results section of paper.

### 3.1 Representation in the Literature: Countries and Population Demographics

**FIGURE 2:**
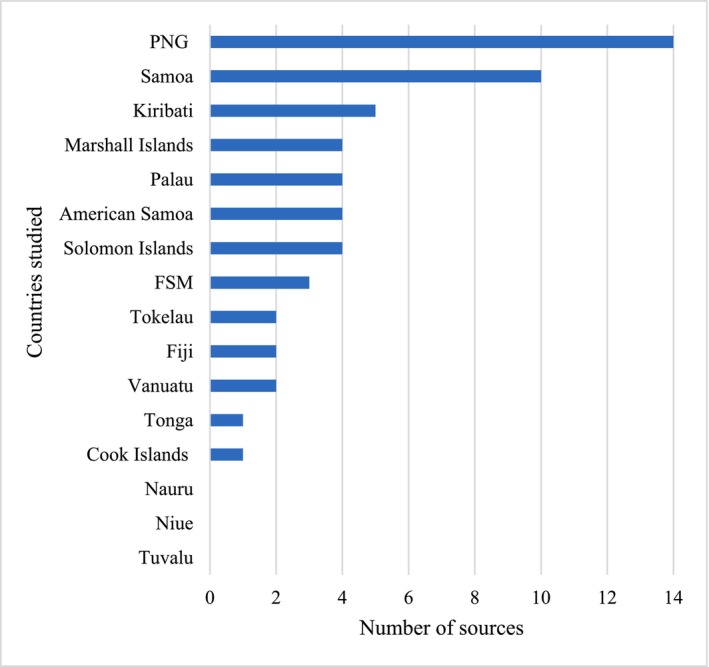
Countries represented in the literature shows the number of relevant sources identified per country.

Studies published in peer‐reviewed journals made up a majority (*n* = 41, 80%) of captured sources, and a further seven gray literature and three PhD dissertations were identified (Table 1). The most frequently represented nations in the literature included PNG (*n* = 14, 27%) and Samoa (*n* = 10, 20%). Kiribati, RMI, Palau, Solomon Islands, American Samoa, and FSM were identified in 3–5 studies each, while dietary intakes in Tokelau, Fiji, Vanuatu, Cook Islands, and Tonga were reported in less than three sources each, respectively. No quantitative macro‐ or micronutrient dietary intake data for Nauru, Niue, or Tuvalu was identified (Figure 2). Most sources reported the dietary intake of adults (*n* = 36, 70%). Dietary intake of adolescents, children, and infants was the focus of investigation in 14 (27%) of identified sources (Binden 1994; Choy et al. 2017, 2022, 2018; Crittenden and Baines 1986; Englberger et al. 2005; Ferro‐Luzzi et al. 1975; Gammino 2001; Gibson et al. 1991; Goris et al. 2018; Hou 2016; Novotny et al. 2022; Pobocik et al. 2000; Thompson et al. 2020; Tyson 1987) and included in several large population cohort studies (Murai 1954; Schultz et al. 2007; Stanhope et al. 1981).

Dietary intake of pregnant and lactating women, and non‐pregnant/non‐lactating women of reproductive age (WRA) was reported in Fiji, Vanuatu, RMI, PNG, Kiribati, Samoa, Palau, Solomon Islands (Arslanian et al. 2020; Goris et al. 2019; Green et al. 2021; Hedges et al. 2009; Kaufer et al. 2010; Murai 1954; Norgan et al. 1974; Pobocik et al. 2000; Schultz et al. 2007; Vogliano et al. 2020, 2021). Several sources specifically investigated nutrition in WRA who were not pregnant or lactating, and two sources studied the dietary intake of pregnant women (Arslanian et al. 2020; Pobocik et al. 2000). Most sources that included dietary intake measurements for sub‐samples of WRA, pregnant and non‐pregnant women were peer‐reviewed journal articles (Arslanian et al. 2020; Goris et al. 2019; Green et al. 2021; Hedges et al. 2009; Norgan et al. 1974; Pobocik et al. 2000; Vogliano et al. 2020, 2021), with one doctoral thesis (Murai 1954) and one gray literature source, the Fiji National Nutrition Survey (Schultz et al. 2007).

### 3.2 Macronutrients and Micronutrients Reported

**FIGURE 3:**
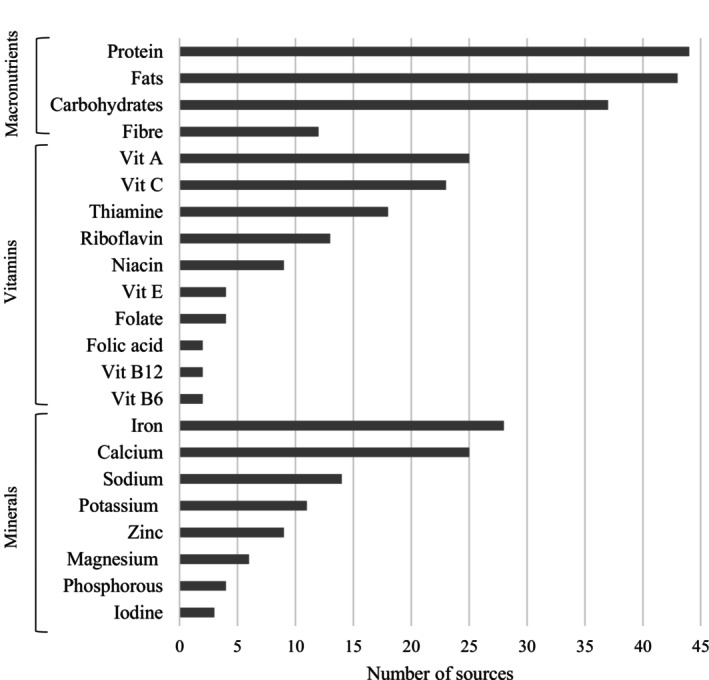
Nutrients reported shows the number of sources reporting macro‐ and micronutrients among PICTs in the captured literature.

Dietary intake of micronutrients was disproportionately reported among the literature. More than half of the studies (*n* = 29, 57%) captured dietary intake data of both macro and micronutrients, while only five papers researched solely micronutrient intake. A variety of vitamins and minerals were studied in the literature, with iron, vitamin A, calcium and vitamin C among the most frequently investigated micronutrients (Table 1). Dietary intake of thiamine, sodium, vitamin B12, potassium, zinc, riboflavin, niacin, magnesium, folate, folic acid (synthetic folate), vitamin E, phosphorous and iodine were investigated, but were reported in less than 30% of identified literature (Figure 3). Macronutrients were reported in (*n* = 46, 90%) of the captured literature, with protein the most frequently studied macronutrient. Intake of phytate, an anti‐nutrient, was also reported in one source (Gibson et al. 1991). Energy (*n* = 45, 88%), whole foods or food groups (*n* = 38, 75%) were often reported alongside nutritional intake to provide further context to the dietary patterns of the population.

### 3.3 Dietary Assessment Methods

Methodology for dietary assessment, data collection and analysis varied significantly across the captured literature. The most utilized dietary assessment methods included 24‐h recall surveys (*n* = 27; 53%), food frequency questionnaires (FFQ; *n* = 14; 27%), and food records (*n* = 13; 25%) (Table 1). Studies often used a combination of these methods to estimate both quantitative intake and dietary patterns within a population (Badcock et al. 1993; Eme et al. 2019, 2020; Englberger et al. 2005; Gould et al. 2013; Hodge et al. 1996a, 1996b; Hou 2016; Schultz et al. 2007; Stanhope et al. 1981; Troubat et al. 2020); however, application of dietary assessments differed greatly depending on setting. Food records conducted by researchers (via direct observation) were conducted for shorter periods (3–7 consecutive days) (Eme et al. 2020; Ferro‐Luzzi et al. 1975; Murai 1954; Norgan et al. 1974; Stanhope et al. 1981; Ulijaszek 1992; Umezaki et al. 1999), while food records kept by participants often had a longer duration (14‐days) (Eme et al. 2019; Hou 2016; Troubat et al. 2020, 2021). Discretionary intake of salt measurements was used by Goris et al. (2018, 2019) to estimate iodine consumption among non‐pregnant women and children in the Gulf Province of PNG.

Findings from Household Income and Expenditure Surveys (HIES) were analyzed to present approximate national dietary intake for several Pacific nations (Hou 2016; Imhoff‐Kunsch et al. 2019; Martyn et al. 2015; SPC 2022b; Reeve et al. 2019; Troubat et al. 2020, 2021; Troubat and Sharp 2021). However, the methods employed to collect dietary intake for national HIES reports differed between PICTs. Some HIES used prospective food records (Troubat et al. 2020, 2021; Hou 2016; Reeve et al. 2019) or modified diet history (SPC 2022b; Troubat and Sharp 2021), while others used food expenditure as a proxy measure for dietary intake (Imhoff‐Kunsch et al. 2019; Martyn et al. 2015; Reeve et al. 2019). Macro and micronutrient intake estimates were commonly determined using food composition tables or food databases. A variety of food databases and nutrient composition tables were utilized by sources to estimate nutrient intake, including the Australian nutrient database, NUTTAB (Hodge et al. 1996a, 1996b), FAO tables and International Network of Food Data Systems (INFOODS) (Vogliano et al. 2020, 2021), New Zealand (NZ) Foodfiles (Hou 2016), the Pacific Tracker (Novotny et al. 2022) and composition tables from Japan (Okuda et al. 1981) and the United States of America (USA) (Galanis et al. 1995; Murai 1954). Pacific Island Food Composition Tables (PIFCT) and later, the Pacific Nutrient Database (PNDB; 2004), were utilized in the literature (Eme et al. 2020; Gammino 2001; Green et al. 2021; Kaufer et al. 2010; Martyn et al. 2015; SPC 2022b; Schultz et al. 2007; Troubat et al. 2021; Troubat and Sharp 2021; Tyson 1987; Ulijaszek 1992; Vogliano et al. 2021), as well as in combination with nutrient composition tables from Japan (Hankin and Dickinson 1972; Konishi et al. 2011), Australia and NZ (Crittenden and Baines 1986; Gould et al. 2013; Green et al. 2021; Hedges et al. 2009; Vogliano et al. 2020), and the USA (Arslanian et al. 2020; Binden 1994; Choy et al. 2017; Crittenden and Baines 1986; DiBello et al. 2009; Galanis et al. 1999; Hankin and Dickinson 1972; Hankin et al. 1970; Reeve et al. 2019; Thompson et al. 2020).

## 4 Discussion

This review revealed a paucity of literature pertaining to macro‐ and micronutrient intake of Pacific Islander peoples. Western Pacific nations were not equally represented in the captured literature, with no dietary intake data identified for Nauruan, Niuean, and Tuvaluan peoples. The disproportionate representation of Pacific nations in the literature may indicate a varied capacity for health surveillance, research, and dissemination of findings (Bissell et al. 2014; Tolley et al. 2016). This is likely because Pacific nations face unique barriers to capturing and reporting health data owing to relatively small, decentralized populations which are spread across hundreds of atolls (Bissell et al. 2014; Craig et al. 2022). The geographical challenges of health research in the Pacific are compounded by technical and logistic barriers, including workforce and funding shortages which result in a limited capacity for health data analysis and reporting (Craig et al. 2022; Tolley et al. 2016; UNICEF 2017).

The ongoing dietary transition and implications of climate change emerged as key sociocultural and environmental drivers of malnutrition in the Pacific. We found the shift away from traditional diets toward more westernized, processed foods was documented as early as the 1970s in Palau (Hankin et al. 1970; Hankin and Dickinson 1972; Labarthe et al. 1973), and continues to be a dominant theme in more recent literature throughout the Pacific (Galanis et al. 1995; Thompson et al. 2020; Vogliano et al. 2021). This transition is closely linked to increased availability and desirability of imported, highly processed and packaged foods, often driven by socioeconomic factors such as income inequality, urbanization, globalization, and changing food systems (Reeve et al. 2022; Sievert et al. 2019). These shifts have influenced dietary behaviors and contributed to rising rates of NCDs such as obesity, diabetes, and hypertension across the region (Badcock et al. 1993; Binden 1994; Choy et al. 2017; Hodge et al. 1996a; Vogliano et al. 2021). Of particular concern, several studies from Samoa, American Samoa, and the Marshall Islands reported these changes in dietary behavior among children and young adults as contributing to malnutrition, stunting and wasting, underweight, and obesity (Binden 1994; Choy et al. 2018; Gammino 2001; Novotny et al. 2022; Thompson et al. 2020). Climate change has exacerbated food insecurity by disrupting local agriculture and subsistence farming, further increasing reliance on imported and packaged foods (Barnett 2020; FAO et al. 2024; Scialabba 2009; Trudinger et al. 2023; WHO Western Pacific Region 2015). Addressing malnutrition in the Pacific therefore requires a holistic understanding of these intersecting influences to inform culturally appropriate and context‐specific nutrition interventions.

Dietary intake of infants and children was the focus of more than a quarter of captured sources in this review, likely a reflection of this population as a nutritionally vulnerable group (Bailey et al. 2015; UNICEF 2024). We found that several studies conducted targeted study of infants and children's nutrition focused on investigating the consumption of key nutrients, such as protein and zinc (Gibson et al. 1991), iodine (Goris et al. 2018), and vitamin A (Englberger et al. 2005). A study by Englberger et al. (2005) reported that around half of all children sampled from a community in Kosrae (FSM) had inadequate intake of vitamin A. In Gulf Province, PNG, researchers identified iodine deficiency among children whose households did not consume iodized salt (Goris et al. 2018). Another study in Wosera District, PNG, reported an association between chronic protein/energy malnutrition and stunting, which was exacerbated by inadequate zinc status (Gibson et al. 1991). These findings are not unique to the Pacific, with deficiency in protein, iodine, vitamin A, and zinc among the most prevalent deficiencies globally (Bailey et al. 2015). Critically, children who are deficient in protein and key micronutrients face a greater risk of stunting and wasting, delayed or impaired cognitive development, and greater vulnerability to infection (Bailey et al. 2015; Black et al. 2013). While several sources included in this review examined the consumption of these specific nutrients, most studies focused on assessing and establishing baseline nutritional status of children in the Pacific, highlighting significant knowledge gaps in this area (Binden 1994; Choy et al. 2017, 2018; Hou 2016; Murai 1954; Novotny et al. 2022; Pobocik et al. 2000; Schultz et al. 2007; Thompson et al. 2020).

Women are recognized as nutritionally vulnerable due to increased requirements associated with menstruation and reproductive health (FAO 2021). Only a few sources included in this review focused on the nutrition of WRA or pregnant women (Arslanian et al. 2020; Goris et al. 2019; Pobocik et al. 2000). Of the studies that did focus on WRA, few acknowledged this population as nutritionally vulnerable or discussed the inherent link between maternal nutrition and infant health outcomes. The ‘developmental origins of adult disease’ hypothesis (Barker hypothesis) proposes adverse influences during gestation can lead to long‐term physiological and metabolic changes of the child, increasing the risk of adverse health outcomes in adulthood (de Boo and Harding 2006). For example, there is a well‐established link between low birth weight (LBW) as a risk factor for NCDs in adulthood (Bianchi and Restrepo 2021; de Boo and Harding 2006; de Mendonça et al. 2020; Nakano 2020). Given this, maternal and pre‐conception nutrition are key determining factors for LBW, and women's nutrition likely plays an essential role in not only influencing maternal and infant health outcomes, but subsequent adult development of disease (da Mota Santana et al. 2021; de Boo and Harding 2006; de Mendonça et al. 2020). More than 20 years ago, Binns et al. (2001) discussed the implications of the Barker hypothesis in the Pacific, calling for further interventions on maternal, prenatal and child nutrition in the region. However, progress on reducing LBW in the Western Pacific has stalled (UNICEF 2018), and rates of adolescent and adult NCDs continue to rise (Peng et al. 2023). Literature identified in this review reveals that inadequate intake of several key micronutrients (iodine, iron, vitamin A, vitamin E, calcium, zinc) is common among WRA and pregnant women, placing this population at a higher risk of maternal and fetal nutritional deficits (Arslanian et al. 2020; Goris et al. 2019; Pobocik et al. 2000). It is critical to note that these findings were limited to available data from women residing in PNG (Goris et al. 2019), Palau (Pobocik et al. 2000) and Samoa (Arslanian et al. 2020). We also observed some variation in reporting the terms used for different micronutrients. For example, ‘folic acid’ and ‘folate’ were used interchangeably in the literature (Zyriax et al. 2018). These terms refer to different forms of vitamin B9; folate being the naturally occurring form found in foods (e.g., dark green leafy vegetables), while folic acid is the synthetic form used in supplements and some fortified products. Future research should ensure that micronutrients are accurately reported as this has important implications for interpretation of findings and subsequent interventions which are essential to not only enhance maternal and foetal nutrition but also reduce the risk of these children developing diseases in adulthood.

Gender roles are likely a key determinant of relationships between food systems and household nutritional wellbeing in the Pacific and should be considered when conducting further research focusing on WRA in the region. Gender roles in the Pacific, specifically women's critical role in maintaining the household, gardening, and cooking, prompted some researchers to capture the dietary intake of WRA (Englberger et al. 2005; Kaufer et al. 2010; Vogliano et al. 2020, 2021). This division of labor is typical across many low‐ and middle‐income countries (LMICs), with women playing a key role in local food systems and household nutrition (Madzorera and Fawzi 2020). In the Western Pacific, women are primarily responsible for continuous maintenance of gardens and food preparation (Georgeou et al. 2022). Women also represent more than 70% of stallholders selling surplus produce at markets in some Pacific nations, supporting household income security (Georgeou et al. 2022). In the captured literature, research methods for three studies in the Solomon Islands (Vogliano et al. 2020, 2021) and FSM (Kaufer et al. 2010) were informed by regional gender roles. Dietary assessments focused on women in the community, given they were primarily responsible for food preparation and gardening in their household (Kaufer et al. 2010; Vogliano et al. 2020, 2021). Another study captured by our review included an assessment of diet (specifically vitamin A consumption) among children and caretakers (Englberger et al. 2005). Interestingly, Englberger et al. (2005) reported that all but one of the participating caretakers were biological or adoptive mothers, emphasizing the role of women as the primary caretaker and as an influential figure in household diet (Englberger et al. 2005). Despite the nutritionally vulnerable status of women, and their influence on child and household nutrition, our review found women remain an under‐represented population in the literature. Future research in this field should consider women as a focal point to understand more broadly household nutrition and food systems in the Western Pacific.

There appears to be no uniform approach to collecting dietary intake data in the Western Pacific. There is significant methodological variation used to estimate diet, which included 24‐h recall surveys, FFQ, food records, diet history, discretionary intake of salt, or Household Income and Expenditure Surveys (HIES). Overall, 24‐h recalls were the most utilized dietary assessment method in the Western Pacific, which is consistent with methodological approaches used broadly across LMICs (de Quadros et al. 2022). When administered by local researchers, this method is adaptable to specific cultural and community contexts and overcomes low literacy barriers (FAO 2018; Windus et al. 2025). Thus, the common use of 24‐h recall surveys in the Pacific is promising, as this method is considered one of the most appropriate for capturing macro‐ and micronutrient intakes in low‐resource settings (de Quadros et al. 2022; FAO 2018; Windus et al. 2025). To investigate nutrition among larger samples or nationally representative populations, some identified sources utilized dietary intake data captured by HIES (Troubat et. al 2020; Hou 2016; Imhoff‐Kunsch et al. 2019; Martyn et al. 2015; SPC 2022b; Reeve et al. 2019; Troubat et al. 2021; Troubat and Sharp 2021). National demographic and health surveys, like HIES, are increasingly being used to estimate nutrient intake on a population level in many LMICs (Tang et al. 2022). A strength of HIES is that the method enables the collection of a variety of socioeconomic and demographic data in addition to nutrient data from a nationally representative population (FAO 2018; Tang et al. 2022), which is critical for understanding malnutrition drivers and in the development of any interventions. Surveys like these also offer a useful and inexpensive opportunity to identify consumption patterns, food availability and estimate the risk of nutrient deficiency of sub‐population groups (FAO 2018). However, household surveys of dietary intake do not capture the distribution of food consumed by individual family members (FAO 2018). This can lead to systematic over‐estimation of consumption of individuals within the household, particularly for women and children (Tang et al. 2022). Our review identified three sources utilizing HIES data which estimated household consumption by‐proxy of food expenditure (Imhoff‐Kunsch et al. 2019; Martyn et al. 2015; Reeve et al. 2019). In addition to not capturing food distribution within the household, this method is further limited by equating food purchases with consumption (FAO 2018). Despite the limitations of HIES for capturing dietary intake data, sources utilizing these datasets (Imhoff‐Kunsch et al. 2019; Martyn et al. 2015; Reeve et al. 2019) still provide us with valuable estimates of nutrient consumption for Pacific populations. The considerable variation in nutrition research methodologies and reporting practices across the Pacific presents a significant barrier to comparing dietary intakes between populations. Establishing standardized methods throughout the region could help to improve the reliability and comparability of findings, strengthening the evidence base for regional nutrition policy development.

Another major limitation for understanding nutrient intake among Pacifika peoples has been a lack of available data regarding the nutrient composition of foods unique to the Pacific. In low‐resource settings, it is common for food composition information from neighboring countries or publicly available regional sources to be used to inform intake analysis, even though such information may not be relevant for a neighboring context (FAO 2018). For example, the earliest studies captured by our review (published in the 1950s–1970s) relied on estimates from the United States Department of Agriculture (USDA) food composition tables. This information was supplemented with some laboratory analysis of samples collected from the Pacific to estimate the nutrient composition of foods consumed by Pacific Islander peoples (Ferro‐Luzzi et al. 1975; Hankin and Dickinson 1972; Murai 1954). During the 1980s–1990s researchers began using more region‐specific food tables informed by previous studies in the Pacific (Binden 1994; Crittenden and Baines 1986; Okuda et al. 1981; Tyson 1987), and in 1983, the Pacific Island Food Composition Tables (PIFCT) were initially released. The PIFCT provided an overview of estimated nutrient composition of Pacific Island foods, the culmination of decades of nutrition research largely driven by the Pacific Community (SPC) and the University of the South Pacific (Dignan et al. 2004). However, these resources were only widely adopted following the publication of reviewed tables by the UN Food and Agriculture Association (FAO) in 2004 (Dignan et al. 2004), and have since played a significant role in informing nutrition research in the Pacific (Arslanian et al. 2020; Binden 1994; Choy et al. 2017; DiBello et al. 2009; Galanis et al. 1999; Gammino 2001; Gould et al. 2013; Green et al. 2021; Hedges et al. 2009; Kaufer et al. 2010; Konishi et al. 2011; Martyn et al. 2015; Reeve et al. 2019; Schultz et al. 2007; Thompson et al. 2020). It is important to note that as diets in the Pacific region have changed significantly over the last 20 years (Sievert et al. 2019), these resource tables may not contain information relevant for all foods consumed present‐day (SPC 2021). Another limitation of the PICFT is the absence of edible portion conversion factors and a lack of nutritional data on pre‐prepared dishes (SPC 2021).

The Pacific Nutrient Database (PNDB) was developed in response to some of the shortfalls of the PICFT (SPC 2021). The PNDB provides a breakdown of nutrient composition on a comprehensive list of food items identified by Pacific household surveys from 2012 to 2016, to facilitate comparable analysis of nutrition data among PICTs (SPC 2021). Since the release of the PNDB in 2020, the database was used in six sources captured in our review (Eme et al. 2020; SPC 2022b; Troubat et al. 2020, 2021; Troubat and Sharp 2021; Vogliano et al. 2020). However, several key micronutrients are notably absent from the PNDB, including folate, iodine and vitamin D (SPC 2022a), all of which are critical for optimal fetal and maternal health. Interestingly, these three micronutrients were also the least researched nutrients among the literature captured in our review. The paucity of available information regarding folate, folic acid, iodine, and vitamin D content in local Pacific foods presents a barrier for researchers estimating the consumption of these micronutrients among Pacific populations, especially in the understanding of adverse maternal and fetal health outcomes. While the PNDB is an essential tool supporting a unified approach to nutrient analysis of Pacific Island foods, the inclusion of several micronutrients would enable more accurate estimation and comparison of dietary intake between Pacific populations.

## 5 Conclusion

This review found a scarcity of literature reporting macro‐ and micronutrient intake in PICTs. Despite the growing body of research on Pacific nutrition, data on dietary intake among nutritionally vulnerable groups such as women and children remain limited. This information gap presents challenges to assessing dietary adequacy and developing targeted interventions to improve nutrition among these groups and the broader population. This review offers a consolidated view of the evidence base, synthesizing available literature on dietary intakes across 16 PICTs into a single, accessible table. This resource provides policy‐makers and researchers with a practical tool to quickly assess what data exists for each country, identify under‐represented populations and nutrients, and prioritize areas for future research and intervention. Encouragingly, our study revealed that research is increasingly utilizing Pacific nutrient databases to inform context‐specific analysis of nutrient composition. While this is a positive trend observed in research, several micronutrients (folate, folic acid, iodine, vitamin D) are notably absent from Pacific nutrient composition databases. These findings offer a valuable evidence base that SPC and national governments can leverage to guide the expansion of the PNDB, supporting more accurate dietary assessments to inform policies, dietary guidelines and nutrition initiatives to address ongoing challenges in Pacific Island Countries and Territories.

## Author Contributions

**Eliza Kitchener:** conceptualization (supporting), data curation (lead), formal analysis (lead), investigation (supporting), methodology (lead), writing – original draft (lead), writing – review and editing (equal). **Rachael Thurecht:** conceptualization (supporting), data curation (supporting), formal analysis (supporting), methodology (supporting), writing – original draft (supporting), writing – review and editing (supporting). **Alannah Connors:** data curation (supporting). **Barnaby Dixson:** data curation (supporting), methodology (supporting), supervision (supporting). **Georgia Kafer:** conceptualization (lead), data curation (supporting), formal analysis (supporting), funding acquisition (lead), methodology (supporting), project administration (lead), supervision (lead), writing – original draft (equal), writing – review and editing (lead).

## Funding

This work was funded by the Research Training Program scholarship (Australian Government).

## Conflicts of Interest

The authors declare no conflicts of interest.

## Data Availability

The data that support the findings of this study are available from the corresponding author upon reasonable request.
