# Correction to “Nutrient Intake Evidence in the Pacific: A Scoping Review of Research Coverage, Challenges, and Opportunities”

**DOI:** 10.1002/fsn3.72283

**Published:** 2026-08-20

Kitchener, E. , R. Thurecht , A. Connors , B. Dixson , and G. Kafer . 2026. “Nutrient Intake Evidence in the Pacific: A Scoping Review of Research Coverage, Challenges, and Opportunities.” Food Science & Nutrition 14, no. 1: e71360. 10.1002/fsn3.71360.41473398 PMC12745662

The last name of the third author was misspelled as “Connors.” The correct author name is Alannah Konners.

The online version of this article has been corrected accordingly.

We apologize for this error.

